# Investigation of Rare Single-Nucleotide *PCDH15* Variants in Schizophrenia and Autism Spectrum Disorders

**DOI:** 10.1371/journal.pone.0153224

**Published:** 2016-04-08

**Authors:** Kanako Ishizuka, Hiroki Kimura, Chenyao Wang, Jingrui Xing, Itaru Kushima, Yuko Arioka, Tomoko Oya-Ito, Yota Uno, Takashi Okada, Daisuke Mori, Branko Aleksic, Norio Ozaki

**Affiliations:** Department of Psychiatry, Nagoya University Graduate School of Medicine, Nagoya, Aichi, Japan; United Graduate School of Child Development, Osaka University, JAPAN

## Abstract

Both schizophrenia (SCZ) and autism spectrum disorders (ASD) are neuropsychiatric disorders with overlapping genetic etiology. *Protocadherin 15* (*PCDH15*), which encodes a member of the cadherin super family that contributes to neural development and function, has been cited as a risk gene for neuropsychiatric disorders. Recently, rare variants of large effect have been paid attention to understand the etiopathology of these complex disorders. Thus, we evaluated the impacts of rare, single-nucleotide variants (SNVs) in *PCDH15* on SCZ or ASD. First, we conducted coding exon-targeted resequencing of *PCDH15* with next-generation sequencing technology in 562 Japanese patients (370 SCZ and 192 ASD) and detected 16 heterozygous SNVs. We then performed association analyses on 2,096 cases (1,714 SCZ and 382 ASD) and 1,917 controls with six novel variants of these 16 SNVs. Of these six variants, four (p.R219K, p.T281A, p.D642N, c.3010-1G>C) were ultra-rare variants (minor allele frequency < 0.0005) that may increase disease susceptibility. Finally, no statistically significant association between any of these rare, heterozygous *PCDH15* point variants and SCZ or ASD was found. Our results suggest that a larger sample size of resequencing subjects is necessary to detect associations between rare *PCDH15* variants and neuropsychiatric disorders.

## Introduction

Schizophrenia (SCZ) and autism spectrum disorders (ASD) are neurodevelopmental in origin. While SCZ and ASD are regarded as separate clinical entities, etiological, clinical, and genetic overlap between them have been discovered [1,2]. Genetic factors make substantial contributions to the etiology of both conditions; heritability is estimated to be a minimum of 80% for each [3]. Thousands of trait- and disease-associated common genetic variants confer increased risk of developing either condition [4,5,6], however, they may explain less than half of the total variation in risk of SCZ [7,8] and ASD [9]. Recent studies suggest that rare copy-number variants (CNVs) and rare single-nucleotide variants (SNVs) may explain additional disease risk or trait variability [10,11,12,13,14,15]. A significant excess of rare, disruptive SNVs has been detected in those genes that have previously been implicated as candidate risk genes for SCZ and/or ASD [16,17,18]. Thus, deep sequencing of candidate genes might be a good way for elucidating the pathogenesis of these neuropsychiatric disorders [19].

*PCDH15* is a member of the largest group in the cadherin superfamily that involved in generating neural diversity for neuronal differentiation and synapse formation [20]. *PCDH15* is primarily recognized as a gene that forms tip-link filaments in sensory hair cells and associated with Usher syndrome type 1F (OMIM 602083) [21]. Notably, more than 20% of these patients exhibit neuropsychiatric symptoms [22]. A GWAS identified *PCDH15* as relevant to neurocognitive processes [23]. In mice, *PCDH15* is expressed throughout the brain and central nervous system (CNS) during embryogenesis [24], and influences serotonin transporter expression in the adult CNS [25]. Rare, exonic CNVs in *PCDH15* were recently identified in ASD [26] and bipolar disorder (BD) patients [27,28]. These findings strongly suggest that *PCDH15* is a promising candidate risk gene for neuropsychiatric disorders because several neuropsychiatric disorders including SCZ, ASD, and BD share genetic risk factors [3,4,5,6,13,27,29,30]. To our knowledge, however, no published study has focused on rare *PCDH15* variants in cases of neuropsychiatric disorders.

Our hypothesis was that rare *PCDH15* variants might confer susceptibility to neuropsychiatric pathogenesis. To increase statistical power and detect shared risk, we combined SCZ and ASD samples in a study cohort [6,31,32]. First, we performed targeted-region sequencing of *PCDH15* coding exons in 562 Japanese patients; we then conducted single-variant association analysis in an independent case-control set comprising 4,013 samples to identify putative variants with large effect.

## Materials and Methods

### Study samples

Two independent Japanese sample groups were used in this study. For the targeted-resequencing discovery cohort, 370 SCZ (mean age ± SD, 49.7 ± 14.8 years; 53.0% male) and 192 ASD (mean age ± SD = 16.3 ± 8.4 years; 77.6% male) individuals participated. For genetic association analysis, the case control sample set comprised 1,714 SCZ (46.3 ± 15.1 years; 51.2% male), 382 ASD (19.6 ± 10.7 years; 77.8% male), and 1,917 control subjects (44.7 ± 14.7 years; 55.3% male). All subjects were unrelated, living on the mainland of Japan, and self-identified as Japanese. All patients fulfilled the criteria listed in *Diagnostic and Statistical Manual of Mental Disorders*, Fifth Edition (DSM-5) for SCZ or ASD. Healthy control subjects were selected from the general population and had no history of mental disorders based on questionnaire responses from the subjects themselves during the sample inclusion step. The study was explained to all participants and/or their parents both verbally and in writing. Written informed consent was obtained from the participants and from the parents of the patients under 20 years old. All procedures performed in this study involving human participants were approved by the Ethics Committee of the Nagoya University Graduate School of Medicine and conducted in accordance with the 1964 Helsinki declaration and its later amendments or comparable ethical standards.

### Sample preparation

DNA was extracted from peripheral blood or saliva from each SCZ, ASD, and control participant. For DNA extraction, we used the QIAamp DNA Blood Kit or Tissue Kit (Qiagen Ltd. Hilden, Germany). The quantity of extracted DNA was estimated using the Qubit^®^ dsDNA BR Assay Kit (Life Technologies, Carlsbad, CA, USA) on a Qubit^®^ 2.0 Fluorometer (Life Technologies, Carlsbad, CA, USA) following the manufacturer’s recommended protocol.

### Library preparation and resequencing

The next-generation sequencing technology on the Ion Torrent PGM^™^ was used to resequence the *PCDH15* coding regions (Ensembl Transcript ID: ENST00000320301; 1995 amino acids) via the protocols described in the Ion AmpliSeq^™^ Library Preparation User Guide (Thermo Fisher Scientific, Rev.5; MAN0006735), Ion PGM^™^ Template OT2 200 Kit (Thermo Fisher Scientific, Rev. 5; MAN0007220), and Ion PGM^™^ Sequencing 200 Kit (Thermo Fisher Scientific, Rev. 3; MAN0007273). After target-specific PCR amplification, amplicons were purified and pooled. Libraries were then prepared to obtain 200-bp PCR fragments flanked by adaptor and barcode sequences; these sequences allowed sequencing and sample identification respectively. The concentration of each library was determined with the Ion Library TaqMan Quantitation Assay Kit (Thermo Fisher Scientific, Waltham, MA, USA). Amplified libraries were subjected to emulsion PCR and subsequent enrichment for template-positive Ion Sphere^™^ particles (ISPs) with the Ion OneTouch^™^ system (Life Technologies, Carlsbad, CA, USA). ISPs were enriched and sequenced in a 200-bp configuration run using 318 chips (Life Technologies, Carlsbad, CA, USA).

### Data analysis

Sequence reads were run through a data analysis pipeline on the Ion Torrent platform-specific pipeline software, Torrent Suite^™^ version 4.4 (Life Technologies, Carlsbad, CA, USA) to generate sequence reads filtered according to the pipeline software quality-controls and to remove poor signal reads. Reads assembling and variant identification were performed with the Ingenuity Variant Analysis software^™^ (http://www.ingenuity.com/variants) from Ingenuity Systems using Fastq files containing sequence reads and the Ion Ampliseq Designer BED file software to map the amplicons with default parameters, (call quality >20 and read depth >10). Candidate variants were defined as exonic or splice-site variants with allele frequencies of ≤1% in the following six public exome databases: dbSNP Build 139 (http://www.ncbi.nlm.nih.gov/projects/SNP/), the 1000 Genomes Project (http://www.1000genomes.org), NHLBI ESP exomes (http://evs.gs.washington.edu/EVS/), the Human Genetic Variation Database (http://www.genome.med.kyoto-u.ac.jp/SnpDB/), the Exome Aggregation Consortium (http://exac.broadinstitute.org) and the Genebook (http://atgu.mgh.harvard.edu/~spurcell/genebook) [17, 27]. To identify deleterious effects caused by amino acid substitution, Sorting Intolerant From Tolerant (SIFT) [33] and PolyPhen-2 (http://genetics.bwh.harvard.edu/pph2/) [34] were used for *in silico* prediction of functional consequences. Additional clinical variant annotations were obtained from NCBI ClinVar (last accessed July 2015; http://www.ncbi.nlm.nih.gov/clinvar/) [35]. To analyze the potential effect of detected variants on putative splicing regulatory elements as exonic splicing enhancer and exonic splicing silencer, we used Splicing-based Analysis of Variants (SPANR) (http://tools.genes.toronto.edu) [36]. Evolutionary conservation was assessed with Evola ver. 7.5 (http://www.h-invitational.jp/evola/search.html) [37]. *De novo* analysis was performed when DNA samples from parents were available.

Sanger sequencing with the ABI 3130xl Genetic Analyzer (Life Technologies, Carlsbad, CA, USA), and standard methods were used to confirm each candidate variant. Sequence analysis software version 6.0 (Applied Biosystems, Foster City, CA, USA) was used to analyze all sequence data. Primer sequences for validating each variant are available in S1 Table.

### Genetic association analysis

An ABI PRISM 7900HT Sequence Detection System (Applied Biosystems, Foster City, CA, USA) and TaqMan assays with custom probes were used to genotype putative deleterious variants. Custom probe sequences are available in S2 Table. Each 384-microtiter plate contained two non-template controls and two samples with the variant. The reactions and data analysis were performed using Genotyping Master Mix and Sequence Detection Systems, respectively, according to the standard protocols (Applied Biosystems, Foster City, CA, USA). Differences in genotype distribution between cases and controls were tested with one-sided, Fisher’s exact tests.

We computed the effective sample size and statistical power using a web browser program, Genetic Power Calculator developed by Purcell et al. (http://pngu.mgh.harvard.edu/~purcell/gpc/) [38].

## Results

### Variation screening of all *PCDH15* coding exons

Nucleotide sequence data reported have been deposited in the DNA Data Bank of Japan (DDBJ) databases (http://www.ddbj.nig.ac.jp) under the accession number DRA004490.

**Fig 1:**
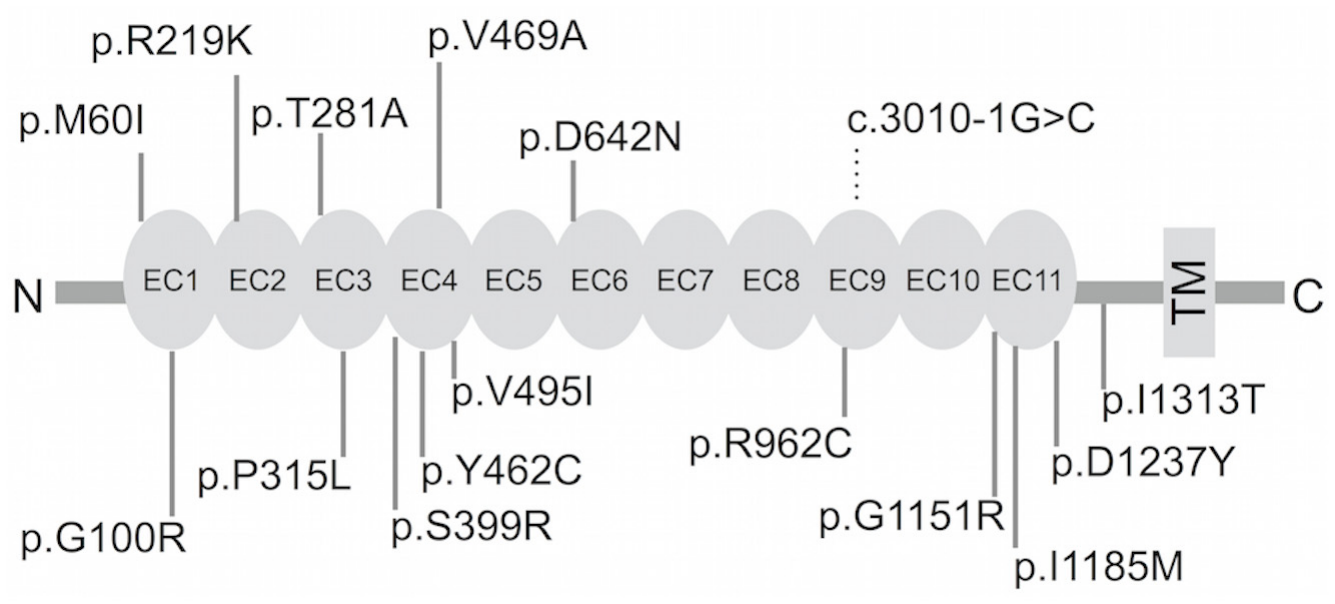
Location for each variant of interest. PCDH15 protein structure is based on NCBI Reference Sequence NP_149045. Each variant was located in the extracellular domain. EC: extracellular cadherin repeat, TM: Transmembrane.

We sequenced *PCDH15* exon and exon-intron boundary in genomic DNA isolated from Japanese patient sample (n = 562). Of 17 SNVs and three indels detected by the Ion Torrent PGM^™^, one SNV and three indels were not validated by Sanger sequencing. In total, we evaluated one splice-site variant and 15 missense variants (Table 1). Analyzing the frequency of rare SNVs in ASD and SCZ individuals, 8.9% of ASD (17/192) and 5.6% of SCZ (17/370) were identified as carriers, pointing to a higher frequency of rare SNVs in ASD (p = 0.037). Nonsense and frameshift variants were not found. Each variant detected was heterozygous. Each of the 15 missense SNVs was located in the coding region of the extracellular domain (Fig 1).

**Table 1:** Rare *PCDH15* SNVs identified in this study.

Chr.	Position (GRCh38)	Ref	Val	Amino Acid changes	Case	Gender	Inheritance status	SIFT	Polyphen-2	dbSNP	1000 Genomes	HGVD^a^	ClinVar	ExAC^b^
10	53840365	A	G	p.I1313T	1 ASD	M	Maternal	Damaging	Possibly Damaging	rs147250420	_	0/3/1	_	2/121412
10	53866650	C	A	p.D1237Y	1 SCZ	F		Damaging	Probably Damaging	rs371278220	_	0/1/299	_	C>T 3/120638
10	53866804	T	C	p.I1185M	1 ASD	F	Paternal	Damaging	Probably Damaging	_	_	0/3/796	_	12/121316
					1 SCZ	M	Paternal							
10	53903293	C	T	p.G1151R	4ASD	F	Paternal	Damaging	Probably Damaging	rs149478475	0.0028	0/21/1084	Likely benign	153/121170
						M	Maternal							
						M	Maternal							
						M	Maternal							
					3 SCZ	1F								
						2M								
10	53959845	C	G	c.3010-1G>C	1 ASD	M	Maternal	_	_	_	_	_	_	_
10	53961877	G	A	p.R962C	1 ASD	M	Paternal	Tolerated	Possibly Damaging	rs201816080	0.0014	0/7/1130	Likely benign	109/121132
					2 SCZ	F								
						M								
10	54090057	C	T	p.D642N	1 SCZ	F		Damaging	Probably Damaging	_	_	_	_	_
10	54183551	C	T	p.V495I	1 SCZ	M		Tolerated	Benign	rs187727835	0.0004	0/1/428	_	1/121400
10	54185168	A	G	p.V469A	1 ASD	M	Maternal	Damaging	Possibly Damaging	_	_	_	_	3/121336
10	54185189	T	C	p.Y462C	1 ASD	M	Maternal	Damaging	Possibly Damaging	rs201284699	0.0004	_	_	11/121346
10	54195793	T	G	p.S399R	2 SCZ	2M		Tolerated	Benign	rs199786639	0.0002	0/4/763	Uncertain significance	31/121404
10	54236864	G	A	p.P315L	3 ASD	M	Maternal	Damaging	Probably Damaging	rs138299477	0.0004	0/13/1096	_	8/121380
						M	Paternal							
						M								
					5 SCZ	3F								
						2M								
10	54317306	T	C	p.T281A	1 ASD	M	Maternal	Tolerated	Possibly Damaging	_	_	_	_	1/121250
10	54329645	C	T	p.R219K	1 ASD	M	Maternal	Tolerated	Possibly Damaging	_	_	_	_	1/121090
10	54378892	C	T	p.G100R	1 ASD	M		Tolerated	Probably Damaging	rs140716525	0.0002	0/3/431	_	16/120962
					1 SCZ	M								
10	54378920	C	T	p.M60I	1 ASD	F	Paternal	Tolerated	Benign	_	_	0/1/367	_	_

*Note*: Amino acid position is based on NCBI reference sequence NP_149045. Chr, chromosome; Ref, reference; Val, variant; M, male; F, female; ASD, autism spectrum disorders; SCZ, schizophrenia; dbSNP, dbSNP build 139 (http://www.ncbi.nlm.nih.gov/projects/SNP/); 1000 Genomes, the 1000 Genomes Project (http://www.1000genomes.org); HGVD, the Human Genetic Variation Database (http://www.genome.med.kyoto-u.ac.jp/SnpDB/); ClinVar, NCBI ClinVar (last accessed July 2015; http://www.ncbi.nlm.nih.gov/clinvar/); ExAC, Exome Aggregation Consortium (http://exac.broadinstitute.org). Rare nonsynonymous SNVs in the Genebook (http://atgu.mgh.harvard.edu/~spurcell/genebook) were not detected in our study.

^a^ homozygous for a minor allele / heterozygote / homozygous for a major allele

^b^ minor allele count / total allele count

Of the 15 missense variants, 12 were predicted to be damaging with *in silico* prediction tools (Table 1). No variants were located in conserved sequences of the cadherin-specific motifs (XEX, DXD, DYE, XDX, and DXNDN) required for calcium binding and rigidification of the extracellular cadherin (EC) domains [39]. Based on the *in silico* predictions, the 15 missense variants were not expected to affect splicing, but one splice-site variant (c.3010-1G>C) was (result not shown).

We were able to determine inheritance status for 16 cases. Among these 16 cases, 10 involved mother-to-son variant transmission, three involved father-to-son transmission, and three others involved father-to-daughter transmission (Table 1). An affected brother shared the p.T281A variant (S1 Fig). An unaffected brother shared the p.G1151R variant, but an unaffected sister of the same patient did not. No *de novo* variants were found in these 16 cases.

We regarded four missense variants (p.R219K, p.T281A, p.V469A, p.D642N) and the splice-site variant (c.3010-1G>C) as novel ones because they were predicted to be damaging or to affect splicing based on *in silico* predictions and because each was not registered in any of the public databases described in the Materials and Methods. p.M60I was included in the association analysis because it was previously detected in a Japanese boy with developmental delay and hearing loss [40], although it was neither classified as damaging in the *in silico* analysis nor absent from the Human Genetic Variation Database. Each of these six SNVs was located in a genomic region that is highly conserved among eight vertebrate species (Table 2). Brief information and results of segregation analysis are in S1 Fig.

**Table 2:** Multiple alignments of amino acid sequences for PCDH15 eight vertebrate homologs.

Variant	Reference	p.M60I	p.R219K	p.T281A	p.V469A	p.D642N
Human	NP_149045	LVDN**M**LIKG	VLRK**R**LNYE	CRPL**T**YQAA	LLQP**V**DREE	LQAT**D**REGD
Chimpanzee	XP_507798.3	LVDN**M**LIKG	VLRK**R**LNYE	CRPL**T**YQAA	LLQP**V**DREE	LQAT**D**REGD
Orangutan	ENSPPYT00000002926	_	VLRK**R**LNYE	CRPL**T**YQAA	LLQP**V**DREE	LQAT**D**REGD
Macaque	XP_001098443.1	LVDN**M**LIKG	VLRK**R**LNYE	CRPL**T**YQAA	LLQP**V**DREE	LQAT**D**REGD
Mouse	ENSMUST00000105426	LVDN**M**LIKG	VLRK**R**LNYE	CRPL**T**YQAA	LLQP**V**DREE	LQAT**D**REGD
Rat	XP_001080000.1	LVDN**M**LIKG	VLRK**R**LNYE	CRPL**T**YQAA	LLQP**V**DREE	LQAT**D**REGD
Chicken	ABC79282.1	LVDN**M**LIKG	VLRE**R**LNYE	CRPL**T**YQAS	LLQP**V**DREA	LQAF**D**REGD
Zebrafish	AAW50924.1	LVEN**M**QING	VLRE**R**LNYE	CKPL**T**YRAS	LLRP**V**DHEE	IQAT**D**REKD

### Genetic association analysis

For our sample set of cases (n = 2,096) and controls (n = 1,917), we computed a statistical power of >80% using the following parameters: disease prevalence of 0.01, observed rare-allele frequency of 0.0021, odds ratio for dominant effect of ≥ 3.59, and type I error rate of 0.0083 (using a Bonferroni correction by a factor of 6, based on the 6 SNVs investigated). An overview and each phenotype of genetic association analysis of the six novel variants are presented in Table 3 and S3 Table. Of the six, four novel SNVs (p.R219K, p.T281A, p.D642N, c.3010-1G>C) remained as singleton observations after genotyping of all cases and controls. We found no statistically significant association for any of the six rare heterozygous point variants in *PCDH15* with case-control analysis. *Post hoc* calculation of statistical power based on a minor allele frequency of 0.00048 (p.M60I, Table 3) revealed that good power accrues for odds ratio ≥ 8.96 or with an increase of the sample size to nearly 20,000 individuals (cases + controls).

**Table 3:** Association analysis of novel rare SNVs.

					Case			Control	
Exon^a^	Ref	Val	Position (GRCh38)	Variant	Genotype count^b^	Minor allele frequency	*P* value^c^	Genotype count^b^	Minor allele frequency
5' side of 23	C	G	10:53959845	c.3010-1G>C	0/0/2085	0	1	0/0/1909	0
16	C	T	10:54090057	p.D642N	0/0/2087	0	1	0/0/1905	0
12	A	G	10:54185168	p.V469A	0/1/2091	0.00024	0.52	0/0/1908	0
8	T	C	10:54317306	p.T281A	0/0/2091	0	1	0/0/1911	0
7	C	T	10:54329645	p.R219K	0/0/2086	0	1	0/0/1915	0
4	C	T	10:54378920	p.M60I	0/2/2090	0.00048	0.19	0/5/1906	0.0013

*Note*: Ref, reference; Val, variant

^a^ Based on ENST00000320301;

^b^ homozygous for a minor allele / heterozygote / homozygous for a major allele;

^C^ P values were calculated by one-tailed Fisher’s exact test

## Discussion

To our knowledge, this is the first study to investigate the contribution of rare *PCDH15* variants to neuropsychiatric disorders and susceptibility to these disorders. We conducted targeted resequencing of coding exons in *PCDH15* for 562 Japanese patients and detected 16 heterozygous SNVs as condition-related candidate genes. More rare SNVs were detected from samples of ASD than those of SCZ. Of these 16 SNVs, five SNVs (p.R219K, p.T281A, p.V469A, p.D642N, c.3010-1G>C) were selected because they were both predicted to be protein-damaging by *in silico* analysis and not registered in public databases or found with a very low frequency in ExAC. p.M60I was selected because it previously implicated in developmental delay [40]. An independent association analysis was then performed with a cohort comprising 2,096 cases and 1,917 controls. Our *a priori* calculation indicated our sample size was appropriately powered to determine statistical significance of SNVs. To assume the odds ratio for dominant effect of rare SNVs would be more than 3.59 seems reasonable according to previous studies that reported odds ratios from 1.88 [41] to 7.1 [32]. Of these six SNVs, four variants (p.R219K, p.T281A, p.D642N, c.3010-1G>C) were not detected in our case control samples, in public databases or found with a very low frequency in ExAC. Although a number of similar studies have identified statistical associations between rare SNVs and SCZ [32,41,42,43], we found no statistically significant association between any of these rare heterozygous *PCDH15* SNVs and either neuropsychiatric disorder.

We find it interesting that all protein-coding SNVs observed in the resequencing cohort were located within the PCDH15 extracellular domain (Fig 1), which may interact with other proteins. Of the 15 protein-damaging SNVs, 12 predicted by *in silico* analysis might change the biological functions of PCDH15. PCDH15 plays an essential role in maintenance of normal retinal and cochlear function [39]. Atypical processing of peripheral sensory inputs plays a crucial role in both SCZ and ASD pathology [44,45,46]. Taken together, deleterious protein changes will induce sensory processing differences contribute to SCZ and ASD symptoms. Notably, splicing misregulation has been implicated in neuropsychiatric disorders [36,47]; c.3010-1G>C also might be a promising candidate for a causal variant in these disease etiopathologies.

In this study, all inheritance statuses were either from apparently unaffected parents or of unknown origin, suggesting variable penetrance (Table 1; S1 Fig). Each candidate variant of maternal origin was transmitted to an affected son; this finding is similar to previous findings [48]. While *de novo* variants have been the focus of research on SCZ and ASD pathogenesis, inherited variants also contribute substantially to these complex diseases [49]. In addition, evolutionary theory predicts that deleterious alleles are likely to be especially rare because of purifying selection [50,51]. Recent large-scale genetic studies report that ultra-rare, private, and inherited-truncating variants in conserved genes are highly enriched in patient populations, especially in genes that closely involved in neurodevelopment [8,17,48,52,53]. The inherited ultra-rare variants (p.R219K, p.T281A, p.D642N, c.3010-1G>C) within highly conserved regions (Table 2) could increase susceptibility to development of a neuropsychiatric disorder.

There are several explanations for our inability to find statistical evidence for a causative role in SCZ and/or ASD for any of these rare *PCDH15* SNVs. First, due to extremely low minor-allele frequencies (< 0.0005) as revealed by the association analysis or to odds ratios lower than expected, we could neither confirm nor dismiss the significance of rare *PCDH15* variants in either neuropsychiatric disorder. *Post hoc* calculations revealed that a larger, higher-powered sample should be sought to reveal relationships between neuropsychiatric disorders and *PCDH15* variants. Secondly, we focused on the shared genetic risk to increase the statistical power in this study. Considering that the burden of rare *PCDH15* was statistically greater in ASD cases than in SCZ, further research may be needed to provide similarities and differences between SCZ and ASD. Thirdly, we focused on the ENST00000320301 transcript, but PCDH15, like many other neuronal proteins, is structurally diversified through the differential inclusion and exclusion of exons. We did not cover the promoter, untranslated regions, or intronic regions of *PCDH15*, which contain potentially disease-relevant regions. Fourthly, the lack of DNA from a sufficient number of patient family members prevented us from monitoring variant segregation. Finally, although 81% of the SNVs (13/16) identified in this study were predicted to be protein-disrupting or splicing-altering based on *in silico* analysis, the exact molecular mechanisms and networks affected by *PCDH15* variants in SCZ and ASD remain unclear. Useful model systems that can address these questions will be needed to assess the impact of the SNVs discovered here.

## Conclusions

We explored the role of rare *PCDH15* SNVs in Japanese SCZ and ASD patients. We found four ultra-rare variants (p.R219K, p.T281A, p.D642N, c.3010-1G>C) that may increase disease susceptibility. No statistically significant association between any rare, heterozygous point *PCDH15* variant and neuropsychiatric disorders was detected. A much larger sample size is needed to elucidate the relevance of rare *PCDH15* variants to neuropsychiatric disorders.

## Supporting Information

S1 Fig Brief information and segregation analysis of cases with six novel variants. The genotypes of the tested individuals are indicated on the lower-side. All comorbidities were diagnosed by experienced psychiatrists according to *Diagnostic and Statistical Manual of Mental Disorders*, *Fifth Edition* (DSM-5) criteria. *Note*: ^1^Autism Spectrum Disorder; ^2^Interectual Disability; ^3^Attention-Deficit/Hyperactivity Disorder; ^4^Tic Disorder; ^5^Epilepsy; ^6^Schizophrenia.
(PDF)

S1 Table Primer sequences for validating each variant. (PDF)

S2 Table Probe sequences for TaqMan SNP assays. *Note*: A TaqMan probe consists with a FAM or VIC dye label on the 5' end, and nonfluorescent quencher (NFQ) on the 3' end.
(PDF)

S3 Table Association results for each phenotype. *Note*: Ref, reference; Val, variant. ^a^ Based on ENST00000320301; ^b^ homozygous for a minor allele / heterozygote / homozygous for a major allele; ^C^ P values were calculated by one-tailed Fisher’s exact test.
(PDF)
