# Supplementary figures and images for: Investigation of Rare Single-Nucleotide PCDH15 Variants in Schizophrenia and Autism Spectrum Disorders
Source: PLoS One. 2016 Apr 8;11(4):e0153224. doi: 10.1371/journal.pone.0153224 (PMC4825995; doi:10.1371/journal.pone.0153224)

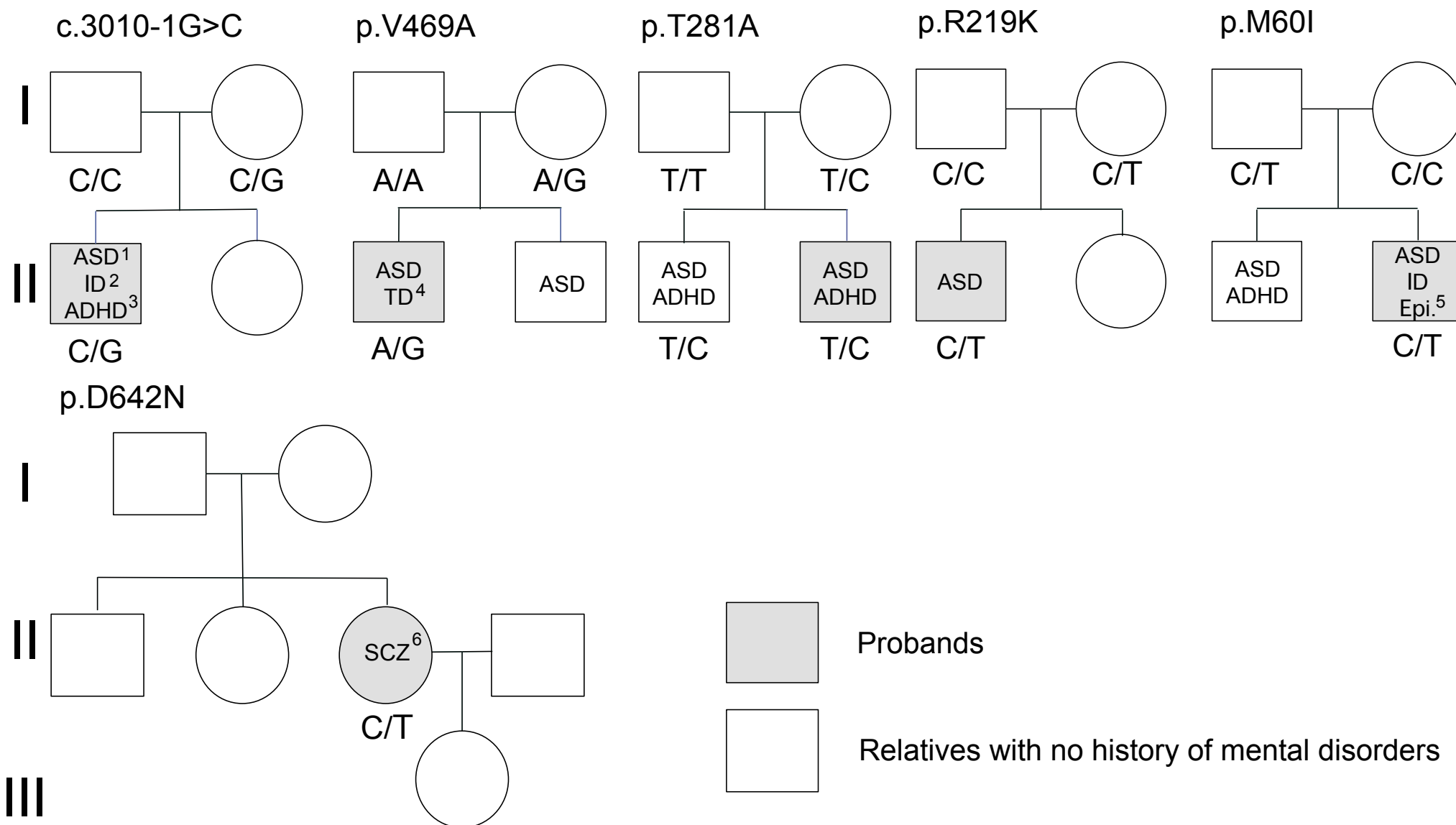

**S1 Fig. Brief information and segregation analysis of cases with six novel variants**

Supplement: S1 Fig — The genotypes of the tested individuals are indicated on the lower-side. All comorbidities were diagnosed by experienced psychiatrists according to Diagnostic and Statistical Manual of Mental Disorders, Fifth Edition (DSM-5) criteria. Note: 1Autism Spectrum Disorder; 2Interectual Disability; 3Attention-Deficit/Hyperactivity Disorder; 4Tic Disorder; 5Epilepsy; 6Schizophrenia. (PDF) [file pone.0153224.s001.pdf]
