# Supplementary material for: Investigation of Rare Single-Nucleotide PCDH15 Variants in Schizophrenia and Autism Spectrum Disorders
Source: PLoS One. 2016 Apr 8;11(4):e0153224. doi: 10.1371/journal.pone.0153224 (PMC4825995; doi:10.1371/journal.pone.0153224)
Supplement: S1 Table — (PDF) [file pone.0153224.s002.pdf]

S1 Table. Primer sequences for validating each variant

Variant	Forward primer (5'-3')	Reverse primer (3'-5')
p.I1313T	TGGTTGCTATTGTAACCAAAGAGCT	CAGAAAGCCCATTGGACTGTCA
p.D1237Y	CTATAAACTGAAAACACTGACCTATGGCTA	GCTCTTCCATAATATGAGGAGATCCTACTT
p.I1185M	CTTCCCATAGTCGTCAGTTGCAATAA	CCATTTCATAAGGGTTATCTTTTCCTGCTT
p.G1151R	CATAATTCCGCATACCTTCACTCTGA	CATGGCATCTAGAAATGGCTTTTGATTTAT
c.3010-1G>C	AGAGACTCCTTTCAAAAATGCCCT	CCAGGCAATCCAATAAGTAAAAACATACAT
p.R962C	CTGTCATCTGTTAAGCCAAATTCTCTATC	TCATTGCACTGTGTTTTAACTTGGTAAC
p.D642N	ACAAACAGCTGAAAGCCTCTGA	GGACATTCTGTGCTATTAGTTTCAAGC
p.V495I	CCCAGGTCTCATGTCTGTATAAACA	TTGTTAAACTGTAAGAGCAAACTCTGTACT
p.V469A	TTTTTCAGTTTTAACCAGACATCTCTTTCA	AAAAGACCCAGAGCTTCACCTTT
p.Y462C	TTTTTCAGTTTTAACCAGACATCTCTTTCA	AAAAGACCCAGAGCTTCACCTTT
p.S399R	TCTATGTCCTTGTCCAGAGCTACTATTC	ACTCCAAAAAGATAGCACGACATATACTTT
p.P315L	TCCTTGGAATTGAGAGAATTTGTTACTGT	TTCATGAAGTATTAGTAATGCGGCTGAA
p.T281A	TCCCATTGGGTTTTAAAACATGCAAAT	GAGATGACTTGGGTCCAATGTTTCT
p.R219K	ACTCACATTAGCTTGGATTATGACAAAGT	TCATCTCTAACTTCCAAGAGGATTAATCCA
p.G100R	TAACGGTTCTCCAGGACACAGA	GTGGATTACTGGGTGTTGATGGAT
p.M60I	GGTGCTGTTCAGGAAAAGCATT	GGTTTAAGAGCATGATCTTTGCTAAAGAAT
