# Supplementary material for: Investigation of Rare Single-Nucleotide PCDH15 Variants in Schizophrenia and Autism Spectrum Disorders
Source: PLoS One. 2016 Apr 8;11(4):e0153224. doi: 10.1371/journal.pone.0153224 (PMC4825995; doi:10.1371/journal.pone.0153224)
Supplement: S2 Table — Note: A TaqMan probe consists with a FAM or VIC dye label on the 5' end, and nonfluorescent quencher (NFQ) on the 3' end. (PDF) [file pone.0153224.s003.pdf]

S2 Table. Probe sequences for TaqMan SNP assays

Variant	Forward primer (5'-3')	Reverse primer (3'-5')	Reporter 1 (5'-3')	Reporter 2 (5'-3')
c.3010-1G>C	CCAGGCAATCCAATAAGTAAAAACATACAT	GGCTCCCCATCATCAAAAGCA	VIC-CCACCAACTTAAAAAG-NFQ	FAM-CCACCAAGTTAAAAAG-NFQ
p.D642N	TCAATTACTTGATAATTGAATTATGTTTTCTCTC	CAATGGCATATGTTATTGAGTCTCCCT	VIC-TGAAGGCAACTGATCGAG-NFQ	FAM-AAGGCAACTAATCGAG-NFQ
p.V469A	GACCCAGAGCTTCACCTTTTTCT	GAAAAGGTGTAAGTTTGCTGTTCTT	VIC-CCCTGTCCACTGGTTG-NFQ	FAM-CCTGTCCGCTGGTTG-NFQ
p.T281A	TGTGCCAAACACTCGTGATTG	CAACTCAGGTATGGCAGCTTG	VIC-CCGTCCACTCACTTAT-NFQ	FAM-CGTCCACTCGCTTAT-NFQ
p.R219K	CTTGGATTATGACAAAGTAGCGAGTCT	ACCTTTGAAATTCCCCTAATGTTGACT	VIC-CATAGTTGAGCCTCTTCC-NFQ	FAM-ATAGTTGAGCTTCTTCC-NFQ
p.M60I	AGTAGAATATGTTTTCAAGTACCTTTCTTTGG	CAGGATCCATCAACACCCAGTAATC	VIC-TGGACAACATGCTGATCA-NFQ	FAM-TGGACAACATACTGATCA-NFQ
