# Supplementary material for: Investigation of Rare Single-Nucleotide PCDH15 Variants in Schizophrenia and Autism Spectrum Disorders
Source: PLoS One. 2016 Apr 8;11(4):e0153224. doi: 10.1371/journal.pone.0153224 (PMC4825995; doi:10.1371/journal.pone.0153224)
Supplement: S3 Table — Note: Ref, reference; Val, variant. a Based on ENST00000320301; b homozygous for a minor allele / heterozygote / homozygous for a major allele; C P values were calculated by one-tailed Fisher’s exact test. (PDF) [file pone.0153224.s004.pdf]

S3 Table. Association results for each phenotype

					ASD			SCZ			Control	
Exon[a]	Ref	Val	Position (GRCh38)	Variant	Genotype Count[b]	Minor allele frequency	*P* value[c]	Genotype Count[a]	Minor allele frequency	*P* value[c]	Genotype Count[a]	Minor allele frequency
5' side of 23	C	G	10:53959845	c.3010-1G>C	0/0/378	0	1	0/0/1707	0	1	0/0/1909	0
16	C	T	10:54090057	p.D642N	0/0/380	0	1	0/0/1707	0	1	0/0/1905	0
12	A	G	10:54185168	p.V469A	0/0/375	0	1	0/1/1716	0.00029	0.47	0/0/1908	0
8	T	C	10:54317306	p.T281A	0/0/381	0	1	0/0/1710	0	1	0/0/1911	0
7	C	T	10:54329645	p.R219K	0/0/381	0	1	0/0/1705	0	1	0/0/1915	0
4	C	T	10:54378920	p.M60I	0/0/381	0	1	0/2/1709	0.00058	0.27	0/5/1906	0.0013
